# The Effect of Sleep on Metabolism, Musculoskeletal Disease, and Mortality in the General US Population: Analysis of Results From the National Health and Nutrition Examination Survey

**DOI:** 10.2196/46385

**Published:** 2023-11-07

**Authors:** Ting Lei, Mingqing Li, Hu Qian, Junxiao Yang, Yihe Hu, Long Hua

**Affiliations:** 1 Department of Orthopedic Surgery Xiangya Hospital Central South University Changsha China; 2 National Clinical Research Center for Geriatric Disorders Xiangya Hospital Central South University Changsha China; 3 Hunan Engineering Research Center of Biomedical Metal and Ceramic Implants Xiangya Hospital Central South University Changsha China; 4 Department of Orthopaedic Surgery Affiliated Hospital of Zunyi Medical University Zunyi China; 5 Department of Orthopedic Surgery The First Affiliated Hospital Zhejiang University Hangzhou China; 6 College of Medicine Zhejiang University Hangzhou China; 7 Department of Orthopedic Surgery The First Affiliated Hospital Xinjiang Medical University Urumqi China; 8 Key Laboratory of High Incidence Disease Research in Xinjiang Ministry of Education Xinjiang Medical University Urumqi China

**Keywords:** sleep duration, mortality, clinical outcomes, threshold effect, National Health and Nutrition Examination Survey

## Abstract

**Background:**

Sleep is an important physiological behavior in humans that is associated with the occurrence and development of various diseases. However, the association of sleep duration with health-related outcomes, including obesity-related factors, musculoskeletal diseases, and mortality because of different causes, has not been systematically reported.

**Objective:**

This study aims to systematically investigate the effect of sleep duration on health-related outcomes.

**Methods:**

Overall, 54,664 participants with sleep information from 8 survey cycles of the National Health and Nutrition Examination Survey (2005-2020) were included in the analysis. Health-related outcomes comprised obesity-related outcomes (ie, BMI, obesity, waist circumference, and abdominal obesity), metabolism-related outcomes (ie, uric acid, hyperuricemia, and bone mineral density [BMD]), musculoskeletal diseases (ie, osteoarthritis [OA] and rheumatoid arthritis [RA]), and mortality because of different causes. The baseline information of participants including age, sex, race, educational level, marital status, total energy intake, physical activity, alcohol consumption, smoking, hypertension, and diabetes was also collected as covariates. Information about the metabolism index, disease status, and covariates was acquired from the laboratory, examination, and questionnaire data. Survival information, including survival status, duration, and cause of death, was obtained from the National Death Index records. Quantile regression models and Cox regression models were used for association analysis between sleep duration and health-related outcomes. In addition, the threshold effect analysis, along with smooth curve fitting method, was applied for the nonlinear association analysis.

**Results:**

Participants were divided into 4 groups with different sleep durations. The 4 groups showed significant differences in terms of baseline data (*P*<.001). The quantile regression analysis indicated that participants with increased sleep duration showed decreased BMI (β=−.176, 95% CI −.220 to −.133; *P*<.001), obesity (odds ratio [OR] 0.964, 95% CI 0.950-0.977; *P*<.001), waist circumference (β=−.219, 95% CI −.320 to −.117; *P*<.001), abdominal obesity (OR 0.975, 95% CI 0.960-0.990; *P*<.001), OA (OR 0.965, 95% CI 0.942-0.990; *P*=.005), and RA (OR 0.940, 95% CI 0.912-0.968; *P*<.001). Participants with increased sleep duration also showed increased BMD (β=.002, 95% CI .001-.003; *P*=.005), as compared with participants who slept <5.5 hours. A significant saturation effect of sleep duration on obesity, abdominal obesity, and hyperuricemia was detected through smooth curve fitting and threshold effect analysis (sleep duration>inflection point). In addition, a significant threshold effect of sleep duration on BMD (*P*<.001); OA (*P*<.001); RA (*P*<.001); and all-cause (*P*<.001), cardiovascular disease−cause (*P*<.001), cancer-cause (*P*=.005), and diabetes-cause mortality (*P*<.001) was found. The inflection point was between 6.5 hours and 9 hours.

**Conclusions:**

The double-edged sword effect of sleep duration on obesity-related outcomes, embolism-related diseases, musculoskeletal diseases, and mortality because of different causes was detected in this study. These findings provided epidemiological evidence that proper sleep duration may be an important factor in the prevention of multisystem diseases.

## Introduction

### Background

A third of our lives is spent sleeping, a crucial activity for maintaining our physiological functions. It is increasingly believed to influence the development of many diseases such as diabetes and death. Some studies have found that sleep deprivation can reduce energy expenditure by slowing metabolism and may also increase the risk of cardiovascular disease (CVD) [1]. Many meta-analyses have shown that short and long sleep durations are associated with all-cause mortality [2-4]. Moreover, sleep may improve musculoskeletal disease at the psychological level [5-8]. It has also been reported that sleep duration is associated with obesity and diabetes [9-11]. However, the problems that cannot be ignored are that there are opposite results reported, differences between different types of studies, diverse target populations and reference groups, insufficient sample size, and other factors; therefore, the benefits of sleep duration on human disease and mortality are still in an uncertain stage.

In addition, different ages, sex, race, education, marital status, body size, lifestyle habits, and the presence of chronic diseases all influenced the outcome variables [12,13]. They are similar to spiderwebs, which have complex connections and restrict or promote each other. At present, few studies include multiple influential factors to establish a prediction model of multiple-disease and multiple-factor mortality and a large sample size to evaluate the correlation between sleep duration and obesity, metabolism-related factors, musculoskeletal disease, and different types of mortalities.

### Objectives

**Figure 1:**
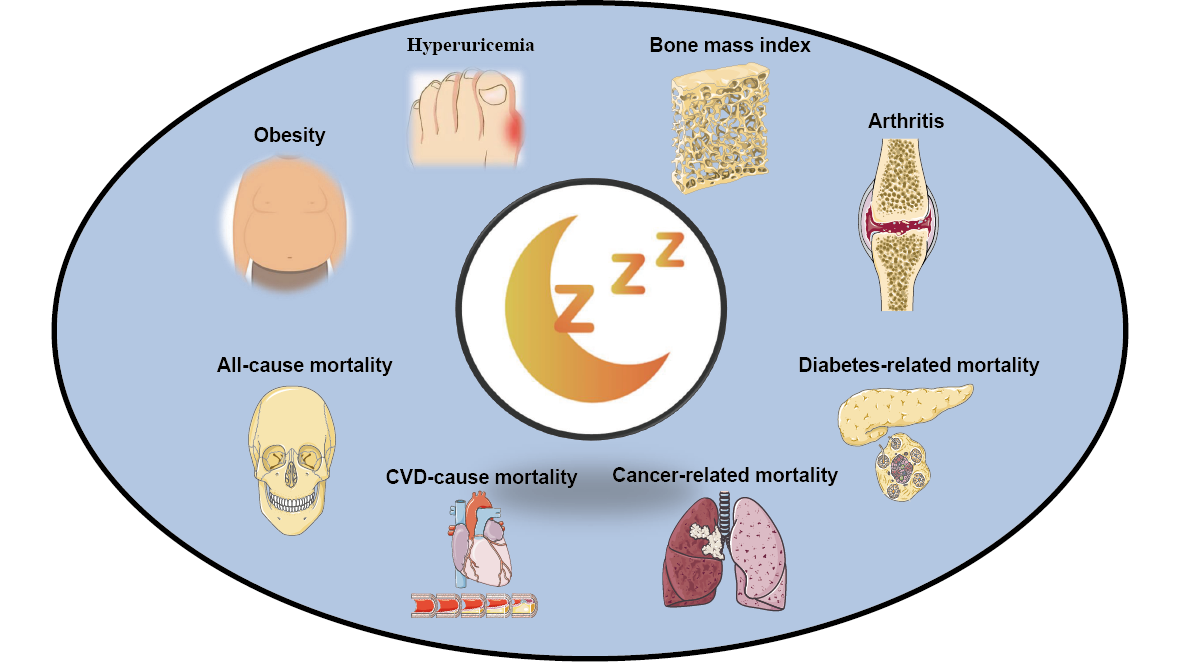
The schematic graph of the relationship between sleep duration and metabolism, musculoskeletal disease, and mortality in the general US population. CVD: cardiovascular diseases.

The US National Health and Nutrition Examination Survey (NHANES) database collected sleep data from participants in the survey program between 2005 and 2020. We used these data to evaluate the complex relationship between sleep duration and obesity metabolism, musculoskeletal disease, and mortality and to elaborate on their essential roles systematically (Figure 1).

## Methods

### Data Source and Population

All data used in this study were acquired from 8 survey cycles (2005-2006, 2007-2008, 2009-2010, 2011-2012, 2013-2014, 2015-2016, 2017-2018, and 2019-2020) of the NHANES program. The NHANES database is designed to survey the nutritional health of the US population every 2 years. It uses a stratified sampling strategy that includes certain groups, such as people aged >60 years and other ethnic groups in the United States. The project includes demographic, inspection, laboratory, and questionnaire data. All data used in this study are available on the NHANES website [14].

### Ethical Considerations

The investigation protocol for this national health program in the United States was reviewed and approved by the National Center for Health Statistics, as one of the departments of the Centers for Disease Control and Prevention. All participants were given an informed consent form outlining the program details, and they would sign it before being included [15]. Furthermore, this study was reviewed and approved by the ethics committee of Xiangya Hospital (202309188).

### Measure of Sleep Duration

In the NHANES program, 8 survey cycles (2005-2020) monitored sleep disorders in people aged >16 years. A detailed measurement method for testing sleep disorders can be found on the website [14]. Sleep information survey collected the enrolled population’s sleep duration and wake duration, including sleep and wake duration on weekdays and weekends. Statistical sleep time was rounded to the nearest half hour. The questions were asked at home by trained interviewers using a computer-assisted personal interview system to ensure data integrity, consistency, and analysis usefulness.

### Obesity Evaluation

BMI assesses obesity, which is obtained as weight/square of height (kg/m^2^), and participants’ obesity status was then determined by BMI, with a BMI of ≥30 being considered obese. For the assessment of abdominal obesity, each participant’s waist circumference (WC; in cm) was obtained for each survey period. Abdominal obesity was defined as WC of >102 cm for men and >88 cm for women. Bone mineral density (BMD) was measured using the dual-energy x-ray absorptiometry method, which has the advantages of high speed, ease of use, and low radiation [16]. Blood uric acid (UA) detection is based on UA oxidation by uricase, under the action of 4-aminophenazone, to produce color products, which the instrument can detect.

### Assessment of Musculoskeletal Diseases

We evaluated musculoskeletal diseases including hyperuricemia (HUA), osteoarthritis (OA), and rheumatoid arthritis (RA). The diagnosis of HUA is determined using the enrolled patient’s serum UA concentration. HUA is diagnosed with serum UA >7mg/mL in men or 6 mg/mL in women. The OA or RA status of participants was obtained through a questionnaire survey. The questions included the following: Have you ever been diagnosed with arthritis? Once the answer was confirmed, they were asked what type of arthritis was diagnosed. Participants were divided into OA, RA, and other groups based on their answers to these 2 questions. A previous study showed an 81% agreement between self-reported OA and clinically diagnosed OA [17].

### Determination of Mortality Outcomes

Mortality information for NHANES program participants is available for download on the National Death Index website, the database used by the National Center for Health Statistics to register death certificate records. The mortality data used in this study were followed up to 2019. National Death Index mortality data included the number of NHANES participants, mortality status, causes of death, and survival time since enlisting in the NHANES program. The cause of death was determined according to the International Statistical Classification of Diseases, 10th edition. The National Institute of Health divided the causes of death into heart disease (054-068) and cancer (019-043). In addition, whether the participants died because of high blood pressure and diabetes was included in the data. In this study, all mortality rates were identified as all-cause mortality, and those related to malignancy were identified as cancer-cause mortality. In addition, deaths related to heart disease or hypertension were identified as CVD-cause mortality. Finally, diabetes-related deaths were identified as diabetes-cause mortality [18].

### Assessment of Covariates

Covariate data were collected and included demographic data such as age, sex, race, education, and marital status. The race consisted of Mexican American, non-Hispanic Black, non-Hispanic White, other Hispanic, and other race. Education level included <9th grade, 9th to 11th grade, high school graduate, some college or Associate of Arts degree, college graduate or above, and others. Marital status included married, widowed, divorced, separated, never married, living with a partner, and others. Data also included BMI, total energy intake, physical activity, alcohol consumption, smoking, high blood pressure, and diabetes. There are subdivisions for each type of data. For example, hypertension is defined as a systolic blood pressure >140 mm Hg and a diastolic blood pressure >90 mm Hg. Diabetes is diagnosed by fasting blood glucose >7.0 mmol/L or the use of hypoglycemic drugs.

### Statistical Analysis

This study aimed to investigate the association between sleep duration and various health-related outcomes using cross-sectional and follow-up data. The analysis between sleep duration and mortality information owing to different causes belongs to the prospective part, as the mortality information was collected after some period of follow-up, whereas the analysis between sleep duration and the other outcomes belongs to the cross-sectional part, as the information of these outcomes was collected at the same time with that of sleep duration and the covariates information. For the prospective part, the Cox quantile regression risk model was used for association analysis between sleep duration and mortality, and the hazard ratio (HR) was used for assessing the effect of sleep duration on mortality-related outcomes. For the cross-sectional part, the quantile regression model was used to analyze the correlation between sleep duration and other outcomes, and the odds ratio (OR) was used for assessing the effect of sleep duration on other health-related outcomes. For continuous variables, β values were used to measure the effect of risk factors on outcome measures. An OR or HR value >1 indicates that the factor is a risk factor. An OR or HR value <1 indicates that the element is protective. A β value of >0 indicates that the factor positively correlates with the outcome measure and vice versa. First, all the participants were equally grouped into 4 quantiles from short to long sleep duration, and the incidence or effective size of clinical outcomes in the first quantile (Q1) was set as the reference. Second, 3 regression models (Cox quantile or quantile regression model) with different covariables were constructed. A nonadjusted model without covariates is first used for the analysis. Model 1 was adjusted for age, sex, race, education, and marital status, and model 2 was adjusted for age, sex, race, education, marital status, BMI, total energy intake, physical activity, smoking, alcohol consumption, hypertension, and diabetes. In addition, baseline data between the 4 groups of participants were compared using the chi-square test (classified data) and the Kruskal-Wallis test (quantitative data). The smooth curve fitting method was used to analyze the nonlinear relationship between sleep duration and these outcomes. According to the results of the smooth curve fitting, the threshold effect analysis was used to investigate whether there were significant differences between the linear model and the 2-piecewise model. A *P* value of <.05 for logarithmic likelihood ratio test was considered to detect the threshold effect of sleep duration on outcomes. A *P* value <.05 was considered statistically significant in all statistical analyses. R software (R Foundation for Statistical Computing) was used for statistical analysis [19].

## Results

### Baseline Data of Participants in Different Sleep Duration

**Figure 2:**
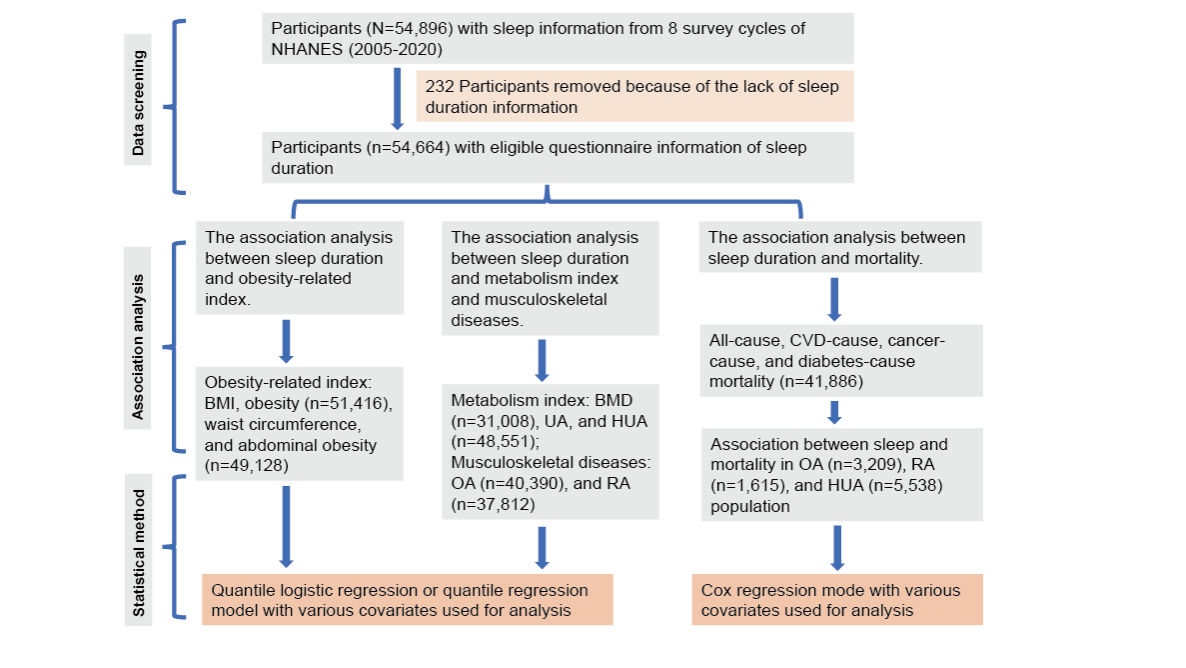
The flow diagram of screening eligible participants from 8 survey cycles (2005-2020) of the National Health and Nutrition Examination Survey (NHANES) program and research structure of association analysis between sleep duration and various clinical outcomes. BMD: bone mineral density; CVD: cardiovascular disease; HUA: hyperuricemia; OA: osteoarthritis; RA: rheumatoid arthritis; UA: uric acid.

A total of 54,896 participants with sleep questionnaire information in the NHANES (2005-2020) were included. After excluding 232 participants with insufficient sleep questionnaire information, 54,664 participants were finally included (Figure 2). Significant differences between the 4 groups were detected in terms of various baseline data, including race composition, educational background, marital status, BMI, total energy intake, physical activity, alcohol consumption, smoking, hypertension, and diabetes (Table 1).

In addition, significant differences between the 4 groups were detected in terms of analyzed outcomes, including obesity-related outcomes, musculoskeletal index, and diseases, as well as mortality because of different causes (Table 2).

**Table 1:** Baseline data comparison of 54,664 participants in different groups divided by sleep duration on the basis of the National Health and Nutrition Examination Survey program (2005-2020; N=54,664; Q1, Q2, Q3, and Q4 represented the intervals of different sleep durations).

		Q1 (n=6862)	Q2 (n=10,524)	Q3 (n=14,198)	Q4 (n=23,080)	*P* value	
Sleep duration (hours)		1-5.5	6-6.5	7-7.5	8-14.5	N/A^a^	
Age (years), mean (SD)		47.76 (17.97)	46.03 (18.21)	45.76 (18.81)	46.65 (21.37)	<.001	
**Sex, n (%)**							<.001
	Male	3452 (50.31)	5345 (50.79)	7111 (50.08)	10,695 (46.34)		
	Female	3410 (49.69)	5179 (49.21)	7087 (49.92)	12,385 (53.66)		
**Race (Hispanic origin), n (%)**							<.001
	Mexican American	827 (12.05)	1618 (15.37)	2223 (15.66)	3940 (17.07)		
	Non-Hispanic Black	2445 (35.63)	2792 (26.53)	2645 (18.63)	4667 (20.22)		
	Non-Hispanic White	2180 (31.77)	3791 (36.02)	5990 (42.19)	9393 (40.70)		
	Other Hispanic	704 (10.26)	1050 (9.98)	1331 (9.37)	2176 (9.43)		
	Other race	706 (10.29)	1273 (12.10)	2009 (14.15)	2904 (12.58)		
**Education level, n (%)**							<.001
	<9th grade	684 (10.60)	883 (9.17)	1154 (9.03)	2344 (11.78)		
	9th-11th grade	1070 (16.58)	1316 (13.67)	1483 (11.60)	2845 (14.30)		
	High school graduate	1669 (25.87)	2235 (23.22)	2764 (21.62)	4602 (23.13)		
	Some college or AA^b^ degree	2095 (32.47)	3026 (31.44)	3670 (28.71)	5685 (28.57)		
	College graduate or above	929 (14.40)	2156 (22.40)	3706 (28.99)	4378 (22.00)		
	Refused	4 (0.06)	4 (0.04)	4 (0.03)	7 (0.04)		
	Do not know	1 (0.02)	6 (0.06)	3 (0.02)	39 (0.20)		
**Marital status, n (%)**							<.001
	Married	2938 (44.83)	5040 (51.28)	7305 (55.84)	10,202 (49.86)		
	Widowed	715 (10.91)	914 (9.30)	1201 (9.18)	2644 (12.92)		
	Divorced	987 (15.06)	1232 (12.54)	1433 (10.95)	2394 (11.70)		
	Separated	283 (4.32)	306 (3.11)	276 (2.11)	473 (2.31)		
	Never married	1138 (17.37)	1677 (17.06)	2057 (15.73)	3396 (16.60)		
	Living with partner	487 (7.43)	649 (6.60)	802 (6.13)	1332 (6.51)		
	Refused	5 (0.08)	10 (0.10)	6 (0.05)	15 (0.07)		
	Do not know	0 (0)	0 (0)	1 (0.01)	4 (0.02)		
BMI (kg/m^2^), mean (SD)		30.09 (7.91)	29.33 (7.19)	28.68 (6.82)	28.49 (7.04)	<.001	
Total energy intake (kcal), mean (SD)		2136.61 (1147.03)	2166.06 (1035.51)	2159.49 (992.91)	2064.69 (978.45)	<.001	
**Physical activity, n (%)**							<.001
	No	3938 (58.14)	6033 (58.04)	8368 (59.77)	14,298 (63.53)		
	Yes	2835 (41.86)	4361 (41.96)	5633 (40.23)	8209 (36.47)		
**Alcohol consumption, n (%)**							N/A
	No	2035 (36.44)	2679 (31.83)	3763 (33.68)	6590 (38.29)		
	Yes	3550 (63.56)	5737 (68.17)	7409 (66.32)	10,620 (61.71)		
**Smoking, n (%)**							<.001
	No	3230 (49.43)	5471 (55.89)	7778 (59.59)	12,107 (58.44)		
	Yes	3304 (50.57)	4318 (44.11)	5274 (40.41)	8609 (41.56)		
**Hypertension, n (%)**							<.001
	No	4095 (59.81)	6980 (66.39)	10,079 (71.05)	15,542 (67.45)		
	Yes	2752 (40.19)	3534 (33.61)	4107 (28.95)	7500 (32.55)		
**Diabetes, n (%)**							<.001
	No	5684 (85.15)	9040 (87.86)	12,434 (89.67)	19,728 (87.29)		
	Yes	991 (14.85)	1249 (12.14)	1433 (10.33)	2873 (12.71)		

^a^N/A: not applicable.

^b^AA: Associate of Arts.

**Table 2:** The comparison of health-related metabolism index, disease information, and mortality information in different groups divided by sleep duration among 54,664 participants in 8 survey cycles of the National Health and Nutrition Examination Survey program (2005-2020; N=54,446; Q1, Q2, Q3, and Q4 represented the intervals of different sleep durations).

			Q1 (n=6862)		Q2 (n=10,524)		Q3 (n=14,198)		Q4 (n=23,080)		*P* value	
Sleep duration (hours)			1-5.5		6-6.5		7-7.5		8-14.5		N/A^a^	
**Obesity, n (%)**												<.001
	No	3713 (57.16)		6142 (61.34)		8647 (64.45)		13,999 (65.14)				
	Yes	2783 (42.84)		3871 (38.66)		4770 (35.55)		7491 (34.86)				
Waist circumference, mean (SD)			100.46 (17.81)		98.87 (17.09)		97.44 (16.38)		97.18 (17.02)		<.001	
**Abdominal obesity, n (%)**												<.001
	No	2600 (42.08)		4368 (45.40)		6173 (47.83)		9641 (47.21)				
	Yes	3579 (57.92)		5254 (54.60)		6734 (52.17)		10,779 (52.79)				
Femur BMD^b^, mean (SD)			1.02 (0.19)		1.02 (0.18)		1.01 (0.18)		1.00 (0.18)		<.001	
Uric acid, mean (SD)			5.50 (1.47)		5.46 (1.45)		5.38 (1.40)		5.37 (1.46)		<.001	
**Hyperuricemia, n (%)**												<.001
	No	4935 (80.43)		7741 (81.78)		10,550 (83.28)		16,747 (82.57)				
	Yes	1201 (19.57)		1725 (18.22)		2118 (16.72)		3534 (17.43)				
**Osteoarthritis, n (%)**												<.001
	No	4267 (86.25)		7101 (88.41)		9680 (88.53)		14,169 (85.99)				
	Yes	680 (13.75)		931 (11.59)		1254 (11.47)		2308 (14.01)				
**Rheumatoid arthritis, n (%)**												<.001
	No	4267 (89.31)		7101 (93.47)		9680 (94.91)		14,169 (92.98)				
	Yes	511 (10.69)		496 (6.53)		519 (5.09)		1069 (7.02)				
**All-cause mortality, n (%)**												<.001
	No	4959 (87.32)		7830 (90.33)		10,064 (92.09)		14,529 (87.47)				
	Yes	720 (12.68)		838 (9.67)		864 (7.91)		2082 (12.53)				
**CVD^c^-cause mortality, n (%)**												<.001
	No	5430 (95.62)		8367 (96.53)		10,645 (97.41)		15,849 (95.41)				
	Yes	249 (4.38)		301 (3.47)		283 (2.59)		762 (4.59)				
**Cancer-cause mortality, n (%)**												<.001
	No	5514 (97.09)		8475 (97.77)		10,703 (97.94)		16,169 (97.34)				
	Yes	165 (2.91)		193 (2.23)		225 (2.06)		442 (2.66)				
**Diabetes-cause mortality, n (%)**												<.001
	No	5581 (98.27)		8579 (98.97)		10,842 (99.21)		16,389 (98.66)				
	Yes	98 (1.73)		89 (1.03)		86 (0.79)		222 (1.34)				

^a^N/A: not applicable.

^b^BMD: bone mineral density.

^c^CVD: cardiovascular disease.

### Correlation Between Sleep Duration and Musculoskeletal Diseases, Obesity, and Metabolism-Related Outcomes

**Figure 3:**
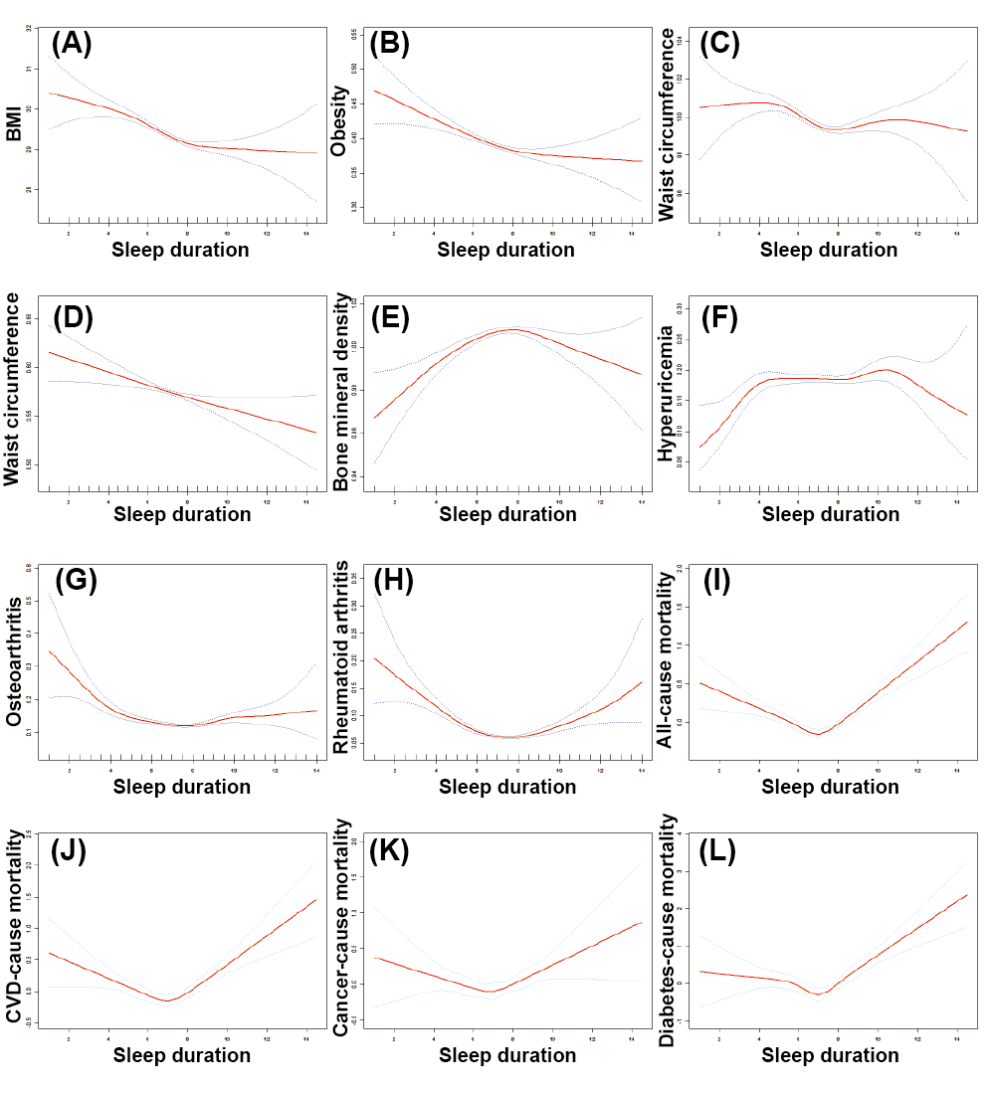
The nonlinear association analysis between sleep duration and (A) BMI; (B) obesity; (C) waist circumference; (D) abdominal obesity; (E) bone mineral density; (F) hyperuricemia; (G) osteoarthritis; (H) rheumatoid arthritis; and (I) Aal-cause, (J) cardiovascular disease (CVD)–cause, (K) cancer-cause, and (L) diabetes-cause mortality among participants in 8 survey cycles of the National Health and Nutrition Examination Survey program (2005-2020) through smooth curve fitting. Note: Except for diabetes-cause mortality, the analysis was adjusted for age, sex, race, educational level, marital status, total energy intake, physical activity, alcohol consumption, smoking, hypertension, and diabetes; the analysis of diabetes-cause mortality was adjusted for age, sex, race, educational level, marital status, total energy intake, physical activity, alcohol consumption, smoking, and hypertension.

**Figure 4:**
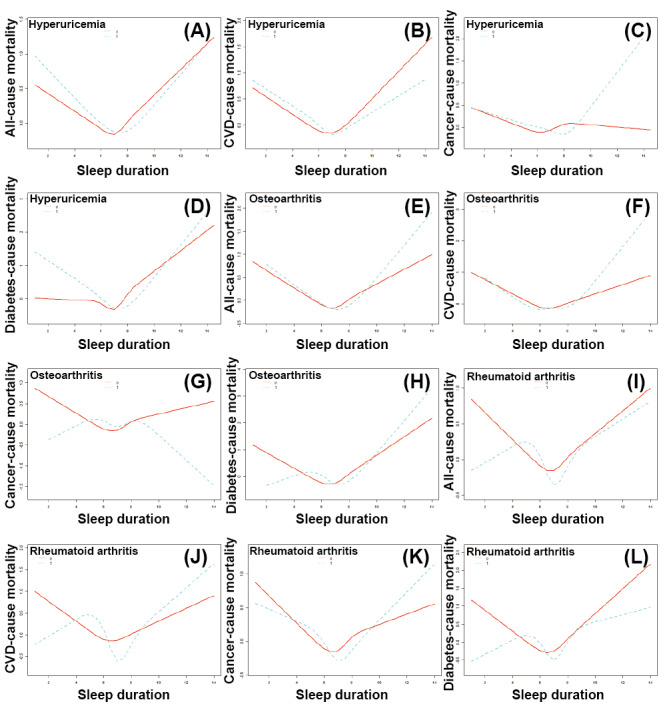
The nonlinear association analysis between sleep duration and mortality of participants experiencing diseases or metabolism disorders through smooth curve fitting: the nonlinear association between sleep and all-cause mortality in 8 survey cycles of the National Health and Nutrition Examination Survey program (2005-2020; (A) all-cause mortality, (B) cardiovascular disease (CVD)–cause mortality, (C) cancer-cause mortality, and (D) diabetes-cause mortality of participants in the US hyperuricemia population; the nonlinear association between sleep and (E) all-cause mortality, (F) CVD-cause mortality, (G) cancer-cause mortality, and (H) diabetes-cause mortality of participants in the US osteoarthritis population; the nonlinear association between sleep and (I) all-cause mortality, (J) CVD-cause mortality, (K) cancer-cause mortality, and (L) diabetes-cause mortality of participants in the US rheumatoid arthritis population. Except for diabetes-cause mortality, the analysis was adjusted for age, sex, race, educational level, marital status, total energy intake, physical activity, alcohol consumption, smoking, hypertension, and diabetes; the analysis of diabetes-cause mortality was adjusted for age, sex, race, educational level, marital status, total energy intake, physical activity, alcohol consumption, smoking, and hypertension.

As shown in Tables 3-6, a negative association between sleep duration and negative association was detected in the whole quantile of all the 3 models. In model 2, the β values of sleep duration on BMI in Q2, Q3, and Q4 were −.308, −.687, and −.931, respectively, and the ORs for obesity in Q2, Q3, and Q4 were 0.924, 0.874, and 0.810, respectively. The β values of sleep duration on WC in Q2, Q3, and Q4 of model 2 were −.593, −1.449, and −1.456, respectively. A negative correlation with abdominal obesity in Q3 (OR 0.898, 95% CI 0.831-0.971) and Q4 (OR 0.871, 95% CI 0.809-0.937) in model 2 was also detected. In contrast, a positive correlation with BMD in Q3 (OR 0.012, 95% CI 0.006-0.008) and Q4 (OR 0.008, 95% CI 0.002-0.013) in model 2 was found, whereas no significant association was detected between sleep duration and UA as well as HUA. In addition, a negative correlation between sleep duration and OA was detected in Q2 (OR 0.839, 95% CI 0.733-0.959), Q3 (OR 0.780, 95% CI 0.686-0.887), and Q4 (OR 0.771, 95% CI 0.683-0.870) in model 2. Similarly, a negative association between sleep duration and RA was detected in the whole quantile of all the 3 models. Subsequently, the nonlinear association using the smooth curve fitting method indicated that sleep duration showed a negative association with BMI, obesity, WC, and abdominal obesity (Figures 3A and 4D). In addition, a n-shaped association between sleep duration and BMD and HUA was detected (Figures 3E and 4F). However, an L-shaped association was observed between sleep duration and OA and RA (Figures 3G and 4H). The threshold effect analysis detected a significant threshold effect of sleep duration on BMI, obesity, and WC (Table 7) as well as on BMD, HUA, OA, and RA (Table 8). The relative inflection points in BMI, obesity, and WC were 8 hours. The relative inflection points in BMD, HUA, OA, and RA were 7.5 hours, 5 hours, 8 hours, and 6.5 hours, respectively. Below the inflection point, negative correlations were detected with BMI, obesity, WC, OA, and RA, whereas a positive correlation was observed between BMD and HUA.

**Table 3:** The association between sleep duration and obesity-related outcomes using the quantile regression method among 51,416 participants in 8 survey cycles of the National Health and Nutrition Examination Survey program (2005-2020). Model 1 was adjusted for age, sex, race, educational level, and marital status; model 2 was adjusted for age, sex, race, educational level, marital status, total energy intake, physical activity, alcohol consumption, smoking, hypertension, and diabetes.

Body index			β (95% CI)	*P* value
**BMI**				
	**Nonadjusted (N=51,416)**			
		1-5.5	0 (0)	N/A^a^
		6-6.5	−.756 (−.979 to .534)	<.001
		7-7.5	−1.407 (−1.618 to 1.196)	<.001
		8-14.5	−1.595 (−1.793 to 1.397)	<.001
	**Model 1 (N=45,818)**			
		1-5.5	0 (0)	N/A
		6-6.5	−.433 (−.658 to .208)	<.001
		7-7.5	−.843 (−1.059 to .627)	<.001
		8-14.5	−1.034 (−1.236 to .831)	<.001
	**Model 2 (N=37,979)**			
		1-5.5	0 (0)	N/A
		6-6.5	−.308 (−.545 to .070)	.01
		7-7.5	−.687 (−.916 to .458)	<.001
		8-14.5	−.931 (−1.146 to .716)	<.001
**WC^b^**				
	**Nonadjusted (N=49,128)**			
		1-5.5	0 (0)	N/A
		6-6.5	−1.587 (−2.129 to 1.044)	<.001
		7-7.5	−3.015 (−3.529 to 2.500)	<.001
		8-14.5	−3.276 (-3.759 to 2.793)	<.001
	**Model 1 (N=43,676)**			
		1-5.5	0 (0)	N/A
		6-6.5	−.860 (−1.386 to .334)	.001
		7-7.5	−1.875 (−2.380 to 1.370)	<.001
		8-14.5	−1.791 (−2.266 to 1.316)	<.001
	**Model 2 (N=37,195)**			
		1-5.5	0 (0)	N/A
		6-6.5	−.593 (−1.143 to .042)	.03
		7-7.5	−1.449 (−1.979 to 0.919)	<.001
		8-14.5	−1.456 (−1.955 to 0.957)	<.001

^a^N/A: not applicable.

^b^WC: waist circumference.

**Table 4:** The association between sleep duration and obesity-related outcomes using the quantile regression method among 51,416 participants in 8 survey cycles of the National Health and Nutrition Examination Survey program (2005-2020).

Obesity outcomes			OR^a^ (95% CI)	*P* value
**Obesity**				
	**Nonadjusted (N=51,416)**			
		1-5.5	1 (0)	N/A^b^
		6-6.5	0.841 (0.789-0.896)	<.001
		7-7.5	0.736 (0.693-0.782)	<.001
		8-14.5	0.714 (0.675-0.755)	<.001
	**Model 1 (N=45,818)**			
		1-5.5	1 (0)	N/A
		6-6.5	0.903 (0.845-0.966)	.003
		7-7.5	0.844 (0.791-0.900)	<.001
		8-14.5	0.799 (0.752-0.849)	<.001
	**Model 2 (N=37,979)**			
		1-5.5	1 (0)	N/A
		6-6.5	0.924 (0.857-0.996)	.04
		7-7.5	0.874 (0.813-0.940)	<.001
		8-14.5	0.810 (0.756-0.867)	<.001
**Abdominal obesity**				
	**Nonadjusted (N=49,128)**			
		1-5.5	1 (0)	N/A
		6-6.5	0.874 (0.819-0.932)	<.001
		7-7.5	0.792 (0.745-0.842)	<.001
		8-14.5	0.812 (0.767-0.860)	<.001
	**Model 1 (N=43,676)**			
		1-5.5	1 (0)	N/A
		6-6.5	0.940 (0.875-1.011)	.10
		7-7.5	0.871 (0.812-0.933)	<.001
		8-14.5	0.856 (0.802-0.914)	<.001
	**Model 2 (N=37,195)**			
		1-5.5	1 (0)	N/A
		6-6.5	0.961 (0.886-1.041)	.33
		7-7.5	0.898 (0.831-0.971)	.007
		8-14.5	0.871 (0.809-0.937)	<.001

^a^OR: odds ratio.

^b^N/A: not applicable.

**Table 5:** The association between sleep duration and bone mineral density, uric acid level, using the quantile regression method among participants in 8 survey cycles of the National Health and Nutrition Examination Survey program (2005-2020). Model 1 was adjusted for age, sex, race, educational level, and marital status; model 2 was adjusted for age, sex, race, educational level, marital status, total energy intake, physical activity, alcohol consumption, smoking, hypertension, and diabetes.

Metabolism index			β (95% CI)	*P* value
**BMD^a^ (g/cm^2^)**				
	**Nonadjusted (N=31,008)**			
		1-5.5	0 (0)	N/A^b^
		6-6.5	.002 (−.005 to .009)	.61
		7-7.5	−.001 (−.008 to .006)	.74
		8-14.5	−.020 (−.026 to −.013)	<.001
	**Model 1 (N=27,966)**			
		1-5.5	0 (0)	N/A
		6-6.5	.004 (−.002 to .010)	.16
		7-7.5	.006 (.001 to .012)	.03
		8-14.5	.001 (−.005 to .006)	.84
	**Model 2 (N=24,485)**			
		1-5.5	0 (0)	N/A
		6-6.5	.006 (−.000 to .012)	.06
		7-7.5	.012 (.006 to .017)	<.001
		8-14.5	.008 (.002 to .013)	.006
**UA^c^(mg/dL)**				
	**Nonadjusted (N=48,551)**			
		1-5.5	0 (0)	N/A
		6-6.5	−.043 (−.089 to .004)	.07
		7-7.5	−.117 (−.161 to −.073)	<.001
		8-14.5	−.134 (−.176 to −.093)	<.001
	**Model 1 (N=43,437)**			
		1-5.5	0 (0)	N/A
		6-6.5	−.014 (−.058 to .030)	.53
		7-7.5	−.060 (−.102 to −.018)	.005
		8-14.5	−.044 (−.084 to .005)	.03
	**Model 2 (N=36,060)**			
		1-5.5	0 (0)	N/A
		6-6.5	.008 (−.037 to .053)	.73
		7-7.5	.008 (−.035 to .052)	.70
		8-14.5	.017 (−.024 to .057)	.43

^a^BMD: bone mineral density.

^b^N/A: not applicable.

^c^UA: uric acid.

**Table 6:** The association between sleep duration and hyperuricemia, osteoarthritis, and rheumatoid arthritis using the quantile regression method among participants in 8 survey cycles of the National Health and Nutrition Examination Survey program (2005-2020).

Diseases				OR^a^ (95% CI)		*P* value
**HUA^b^**						
	**Nonadjusted (N=48,511)**					
		1-5.5	1 (0)		N/A^c^	
		6-6.5	0.916 (0.844-0.994)		.03	
		7-7.5	0.825 (0.763-0.892)		<.001	
		8-14.5	0.867 (0.806-0.933)		<.001	
	**Model 1 (N=43,437)**					
		1-5.5	1 (0)		N/A	
		6-6.5	0.987 (0.906-1.075)		.77	
		7-7.5	0.921 (0.848-1.001)		.05	
		8-14.5	0.949 (0.879-1.025)		.18	
	**Model 2 (N=36,060)**					
		1-5.5	1 (0)			
		6-6.5	1.041 (0.944-1.149)		.42	
		7-7.5	1.059 (0.963-1.164)		.24	
		8-14.5	1.057 (0.967-1.155)		.22	
**OA^d^**						
	**Nonadjusted (N=40,390)**					
		1-5.5	1 (0)		N/A	
		6-6.5	0.823 (0.740-0.915)		<.001	
		7-7.5	0.813 (0.736-0.898)		<.001	
		8-14.5	1.022 (0.932-1.121)		.64	
	**Model 1 (N=40,390)**					
		1-5.5	1 (0)		N/A	
		6-6.5	0.796 (0.709-0.895)		<.001	
		7-7.5	0.705 (0.630-0.788)		<.001	
		8-14.5	0.728 (0.657-0.808)		<.001	
	**Model 2 (N=31,502)**					
		1-5.5	1 (0)		N/A	
		6-6.5	0.839 (0.733-0.959)		.01	
		7-7.5	0.780 (0.686-0.887)		<.001	
		8-14.5	0.771 (0.683-0.870)		<.001	
**RA^e^**						
	**Nonadjusted (N=37,812)**					
		1-5.5	1 (0)		N/A	
		6-6.5	0.583 (0.513-0.664)		<.001	
		7-7.5	0.448 (0.394-0.509)		<.001	
		8-14.5	0.630 (0.564-0.704)		<.001	
	**Model 1 (N=37,812)**					
		1-5.5	1 (0)		N/A	
		6-6.5	0.647 (0.564-0.741)		<.001	
		7-7.5	0.515 (0.450-0.589)		<.001	
		8-14.5	0.587 (0.521-0.660)		<.001	
	**Model 2 (N=29,476)**					
		1-5.5	1 (0)		N/A	
		6-6.5	0.667 (0.572-0.779)		<.001	
		7-7.5	0.561 (0.481-0.654)		<.001	
		8-14.5	0.631 (0.551-0.724)		<.001	

^a^OR: odds ratio.

^b^HUA: hyperuricemia.

^c^N/A: not applicable.

^d^OA: osteoarthritis.

^e^RA: rheumatoid arthritis.

**Table 7:** Threshold effect analysis^a^ of sleep duration on obesity-related outcomes among 37,979 participants in 8 survey cycles of the National Health and Nutrition Examination Survey program (2005-2020).

Obesity-related outcomes			BMI (N=37,979), β (95% CI)		Obesity (N=37,979), OR^b^ (95% CI)		WC^c^ (n=37,195), β (95% CI)		AB obesity^d^ (n=37,195), OR (95% CI)
Effect analysis by one linear model			−0.176^e^ (−0.220 to −0.133)		0.964^e^ (0.950 to 0.977)		−0.219^e^ (−0.320 to −0.117)		0.975^e^ (0.960 to 0.990)
**Effect analysis by 2-fold piecewise model**									
	Effect difference	−0.249^e^ (−0.309 to −0.190)		0.943^e^ (0.925 to 0.961)		−0.407^e^ (−0.546 to −0.269)		0.963^e^ (0.943 to 0.982)	
	Sleep duration<inflection point	0.006 (−0.104 to 0.116)		1.018 (0.983 to 1.054)		0.258^f^ (0.000 to 0.516)		1.008 (0.970 to 1.047)	
	Sleep duration≥inflection point	0.255^e^ (0.114 to 0.397)		1.079^g^ (1.032 to 1.129)		0.666^e^ (0.335 to 0.997)		1.047 (0.997 to 1.099)	

^a^The model used for the threshold effect analysis was adjusted for age, sex, race, educational level, marital status, total energy intake, physical activity, alcohol consumption, smoking, hypertension, and diabetes.

^b^OR: odds ratio.

^c^WC: waist circumference.

^d^AB obesity: abdominal obesity.

^e^*P*<.001.

^f^*P*<.05.

^g^*P*<.01.

**Table 8:** Threshold effect analysis^a^ of sleep duration on bone mineral density (BMD), hyperuricemia, osteoarthritis, and rheumatoid arthritis (RA) among 36,060 participants in 8 survey cycles of the National Health and Nutrition Examination Survey program (2005-2020; N=36,060).

Obesity-related outcomes			BMD (n=24,485), β (95% CI)		Hyperuricemia (N=36,060), OR^b^ (95% CI)		Osteoarthritis (n=31,502), OR (95% CI)		RA (n=29,476), OR (95% CI)
Effect analysis by one linear model			0.002^c^ (0.001 to 0.003)		1.015 (0.997 to 1.033)		0.965^c^ (0.942 to 0.990)		0.940^d^ (0.912 to 0.968)
**Effect analysis by 2-fold piecewise model**									
	Sleep duration<inflection point	0.006^d^ (0.004 to 0.007)		1.139^c^ (1.035 to 1.253)		0.901^d^ (0.871 to 0.932)		0.726^d^ (0.681 to 0.773)	
	Sleep duration≥inflection point	−0.005^d^ (−0.007 to −0.002)		1.002 (0.981 to 1.023)		1.135^d^ (1.070 to 1.203)		1.103^d^ (1.055 to 1.154)	
	Effect difference	−0.010^d^ (−0.014 to −0.006)		0.880^e^ (0.793 to 0.976)		1.259^d^ (1.166 to 1.360)		1.520^d^ (1.385 to 1.668)	

^a^The model used for threshold effect analysis was adjusted for age, sex, race, educational level, marital status, BMI, total energy intake, physical activity, alcohol consumption, smoking, hypertension, and diabetes.

^b^OR: odds ratio.

^c^*P*<.01.

^d^*P*<.001.

^e^*P*<.05.

### Association Between Sleep Duration and Mortality in General Population

As shown in Table 9, a significantly negative correlation between sleep duration and all-cause mortality was found in Q2 (HR 0.844) and Q3 (HR 0.749) of model 2, respectively. Consistently, a significantly negative correlation with CVD-cause mortality in Q2 (HR 0.773) in model 2 was discovered. For cancer-cause mortality, a significant HR in Q2 (HR 0.801) and Q3 (HR 0.798) of model 1 was detected, which was absent in model 2. For diabetes-cause mortality, a significantly negative HR was detected in Q2 (HR 0.654) and Q3 (HR 0.0.562) of model 1 as well as Q3 (HR 0.633) of model 2. The analysis results of smooth curve fitting indicated an L-shaped association between sleep duration and all-cause, CVD-cause, cancer-cause, and diabetes-cause mortality (Figures 3I and 4L). The threshold effect analysis detected a significant threshold effect of sleep duration on all-cause, CVD-cause, cancer-cause, and diabetes-cause mortality, and an inflection point of 6.5 hours was detected (Table 10). When below the inflection point, a significantly protective HR of sleep duration was detected in terms of all-cause, CVD-cause, cancer-cause, and diabetes-cause mortality. However, when above the inflection point, a significant risk HR of sleep duration was detected in terms of all-cause, CVD-cause, cancer-cause, and diabetes-cause mortality.

**Table 9:** The association between sleep duration and hazard ratios for mortality in population using the quantile regression method among 51,416 participants in 8 survey cycles of the National Health and Nutrition Examination Survey program (2005-2020).

		Nonadjusted	*P* value	Model 1	*P* value	Model 2	*P* value
**All-cause mortality, n (%)**		41,886 (100)	N/A^a^	39,501 (100)	N/A	31,419 (100)	N/A
	1-5.5, HR^b^ (95% CI)	1 (0)	N/A	1 (0)	N/A	1 (0)	N/A
	6-6.5, HR (95% CI)	0.752 (0.681-0.831)	<.001	0.800 (0.724-0.884)	<.001	0.844 (0.751-0.949)	.005
	7-7.5, HR (95% CI)	0.651 (0.589-0.718)	<.001	0.708 (0.641-0.783)	<.001	0.749 (0.666-0.842)	<.001
	8-14.5, HR (95% CI)	1.171 (1.076-1.275)	<.001	0.969 (0.888-1.056)	.47	0.975 (0.879-1.081)	.63
**CVD^c^-cause mortality, n (%)**		41,886 (100)	N/A	39,501 (100)	N/A	31,419 (100)	N/A
	1-5.5, HR (95% CI)	1 (0)	N/A	1 (0)	N/A	1 (0)	N/A
	6-6.5, HR (95% CI)	0.781 (0.661-0.924)	.004	0.843 (0.712-0.998)	.047	0.909 (0.745-1.107)	.37
	7-7.5, HR (95% CI)	0.617 (0.520-0.731)	<.001	0.689 (0.579-0.818)	<.001	0.773 (0.632-0.945)	.01
	8-14.5, HR (95% CI)	1.241 (1.076-1.432)	.003	1.010 (0.873-1.169)	.89	1.020 (0.857-1.215)	.82
**Cancer-cause mortality, n (%)**		41,886 (100)	N/A	39,501 (100)	N/A	31,419 (100)	N/A
	1-5.5, HR (95% CI)	1 (0)	N/A	1 (0)	N/A	1 (0)	N/A
	6-6.5, HR (95% CI)	0.756 (0.614-0.931)	.008	0.801 (0.650-0.987)	.04	0.805 (0.632-1.025)	.08
	7-7.5, HR (95% CI)	0.738 (0.604-0.903)	.003	0.798 (0.650-0.978)	.03	0.813 (0.642-1.030)	.09
	8-14.5, HR (95% CI)	1.078 (0.902-1.290)	.41	0.925 (0.771-1.110)	.40	0.964 (0.780-1.192)	.74
**Diabetes-cause mortality, n (%)**		41,886 (100)	N/A	39,501 (100)	N/A	31,419 (100)	N/A
	1-5.5, HR (95% CI)	1 (0)	N/A	1 (0)	N/A	1 (0)	N/A
	6-6.5, HR (95% CI)	0.587 (0.441-0.782)	<.001	0.654 (0.490-0.873)	.004	0.738 (0.525, 1.038)	.08
	7-7.5, HR (95% CI)	0.476 (0.357-0.636)	<.001	0.562 (0.419-0.754)	<.001	0.633 (0.446-0.899)	.01
	8-14.5, HR (95% CI)	0.922 (0.726-1.169)	.50	0.842 (0.661-1.072)	.16	1.072 (0.805-1.429)	.63

^a^N/A: not applicable.

^b^HR: heart rate.

^c^CVD: cardiovascular disease.

**Table 10:** Threshold effect analysis of sleep duration on mortality with different causes among 31,419 participants in 8 survey cycles in the National Health and Nutrition Examination Survey program (2005-2020; N=31,419).

Mortality		All-cause mortality (N=31,419)^a^, HR^b^ (95% CI)	Cardiovascular disease–cause mortality (N=31,419)^a^, HR (95% CI)	Cancer-cause mortality (N=31,419)^a^, HR (95% CI)	Diabetes-cause mortality (n=32,173)^c^, HR (95% CI)
Effect analysis by one linear model		1.036^d^ (1.012-1.060)	1.043^e^ (1.003-1.084)	1.028 (0.980-1.079)	1.018 (0.955-1.084)
**Effect analysis by 2-fold piecewise model**					
	Sleep duration<inflection point	0.847^f^ (0.806-0.889)	0.835^f^ (0.770-0.906)	0.892^e^ (0.804-0.990)	0.813^d^ (0.709-0.933)
	Sleep duration≥inflection point	1.169^f^ (1.129-1.210)	1.188^f^ (1.122-1.258)	1.120^d^ (1.041-1.206)	1.150^d^ (1.050-1.260)
	Effect difference	1.381^f^ (1.285-1.484)	1.422^f^ (1.262-1.603)	1.255^f^ (1.077-1.462)	1.414^f^ (1.161-1.722)

^a^The model used for the threshold effect analysis was adjusted for age, sex, race, educational level, marital status, total energy intake, BMI, physical activity, alcohol consumption, smoking, hypertension, and diabetes.

^b^HR: hazard ratio.

^c^The model used for threshold effect analysis was adjusted for age, sex, race, educational level, marital status, total energy intake, BMI, physical activity, alcohol consumption, smoking, and hypertension.

^d^*P*<.01.

^e^*P*<.05.

^f^*P*<.001.

### Association Between Sleep Duration and Mortality in RA, OA, and HUA Populations

As shown in Table 11, in the HUA population, the HR of all-cause mortality in Q2, Q3, and Q4 was 0.784 (95% CI 0.633-0.972; *P*=.03), 0.669 (95% CI 0.538-0.832; *P*<.001), and 0.786 (95% CI 0.649-0.953; *P*=.01), respectively. The HR of CVD-cause mortality in Q3 was 0.667 (95% CI 0.471-0.944; *P*=.02). The HR of diabetes-cause mortality in Q2 and Q3 was 0.587 (95% CI 0.345-0.996; *P*=.048) and 0.464 (95% CI 0.266-0.810; *P*=.007), respectively. In the OA population, sleep duration was identified as a protective factor for OA in Q3 of all-cause mortality. The HR for all-cause mortality in Q3 was 0.647 (95% CI 0.487-0.861; *P*=.003). In the RA population, sleep duration was identified as a protective factor for RA in Q3 (HR 0.500, 95% CI 0.338-0.742; *P*<.001) of all-cause mortality and Q3 (HR 0.302, 95% CI 0.143-0.638; *P*=.002) of CVD-cause mortality. The analysis results of the smooth curve fitting showed an L-shaped association between sleep duration and mortality in participants with HUA (Figure 4) and a n-shaped association between sleep duration and cancer-cause mortality in the OA population. The threshold effect analysis found a significant threshold effect of sleep duration on all-cause, CVD-cause, cancer-cause, and diabetes-cause mortality in the HUA population, which was also detected for all-cause and CVD-cause mortality in the OA and RA populations as well as diabetes-cause mortality in the OA population. In the OA population, the detected inflection points of sleep duration for all-cause, CVD-cause, and diabetes-cause mortality were 6.5 hours, 8.5 hours, and 7.5 hours, respectively (Table 12).

**Table 11:** The effect of sleep duration on mortality of population experiencing musculoskeletal disorders using the quantile regression method among 5538 participants in 8 survey cycles of the National Health and Nutrition Examination Survey program (2005-2020). The model was adjusted by age, sex, race, educational level, marital status, total energy intake, physical activity, alcohol consumption, smoking, hypertension, and diabetes.

		HUA^a^ population	*P* value	OA^b^ population	*P* value	RA^c^ population	*P* value
**All-cause mortality, n (%)**		5538 (100)	N/A^d^	3209 (100)	N/A	1615 (100)	N/A
	1-5.5, HR^e^ (95% CI)	1 (0)	N/A	1 (0)	N/A	1 (0)	N/A
	6-6.5, HR (95% CI)	0.784 (0.633-0.972)	.03	0.782 (0.591-1.034)	.08	0.956 (0.683-1.337)	.79
	7-7.5, HR (95% CI)	0.669 (0.538-0.832)	<.001	0.647 (0.487-0.861)	.003	0.500 (0.338-0.742)	<.001
	8-14.5, HR (95% CI)	0.786 (0.649-0.953)	.01	0.863 (0.670-1.110)	.25	0.913 (0.675-1.236)	.56
**CVD-cause mortality, n (%)**		5538 (100)	N/A	3209 (100)	N/A	1615 (100)	N/A
	1-5.5, HR (95% CI)	1 (0)	N/A	1 (0)	N/A	1 (0)	N/A
	6-6.5, HR (95% CI)	0.818 (0.583-1.147)	.24	0.772 (0.471-1.266)	.30	0.985 (0.575-1.688)	.96
	7-7.5, HR (95% CI)	0.667 (0.471-0.944)	.02	0.795 (0.492-1.285)	.35	0.302 (0.143-0.638)	.002
	8-14.5, HR (95% CI)	0.804 (0.594-1.088)	.16	1.097 (0.720-1.672)	.67	0.791 (0.477-1.311)	.36
**Cancer-cause mortality, n (%)**		5538 (100)	N/A	3209 (100)	N/A	1615 (100)	N/A
	1-5.5, HR (95% CI)	1 (0)	N/A	1 (0)	N/A	1 (0)	N/A
	6-6.5, HR (95% CI)	0.919 (0.565-1.494)	.73	1.489 (0.778-2.848)	.23	0.817 (0.421-1.584)	.55
	7-7.5, HR (95% CI)	0.796 (0.486-1.303)	.36	1.080 (0.552-2.114)	.82	0.522 (0.246-1.107)	.09
	8-14.5, HR (95% CI)	0.947 (0.610-1.469)	.81	1.211 (0.646-2.272)	.55	0.833 (0.463-1.497)	.54
**Diabetes-cause mortality, n (%)**		5725 (100)	N/A	3345 (100)	N/A	1669 (100)	N/A
	1-5.5, HR (95% CI)	1 (0)	N/A	1 (0)	N/A	1 (0)	N/A
	6-6.5, HR (95% CI)	0.587 (0.345-0.996)	.048	0.763 (0.338-1.723)	.52	0.970 (0.342-2.746)	.95
	7-7.5, HR (95% CI)	0.464 (0.266-0.810)	.007	0.737 (0.323-1.685)	.47	0.396 (0.101-1.552)	.18
	8-14.5, HR (95% CI)	0.661 (0.418-1.046)	.08	0.975 (0.481-1.975)	.94	1.220 (0.490-3.036)	.67

^a^HUA: hyperuricemia.

^b^OA: osteoarthritis.

^c^RA: rheumatoid arthritis.

^d^N/A: not applicable.

^e^HR: heart rate.

**Table 12:** Threshold effect analysis^a^ of sleep duration on mortality with different causes among 3345 participants with osteoarthritis (OA), 1669 participants with rheumatoid arthritis (RA), and 5725 participants with hyperuricemia (HUA) in 8 survey cycles of the National Health and Nutrition Examination Survey program (2005-2020).

Specific population					All-cause mortality			CVD^b^-cause mortality			Cancer-cause mortality			Diabetes-cause mortality
**OA population, n (%)**					3209 (100)			3209 (100)			3209 (100)			3345 (100)
	Effect analysis by one linear model, HR^c^ (95% CI)			1.037 (0.980-1.098)			1.153^d^ (1.052-1.264)			0.978 (0.863-1.109)			1.164 (0.988-1.370)	
	**Effect analysis by 2-fold piecewise model, HR (95% CI)**													
		Sleep duration＜inflection point	0.755^e^ (0.671-0.851)			0.961 (0.855-1.079)			1.003 (0.874-1.152)			0.851 (0.665-1.087)		
		Sleep duration≥inflection point	1.236^e^ (1.140-1.339)			1.778^e^ (1.470-2.150)			0.558 (0.138-2.259)			1.663^e^ (1.288-2.148)		
		Effect difference	1.636^e^ (1.377-1.942)			1.850^e^ (1.424-2.404)			0.556 (0.132-2.339)			1.956^d^ (1.274-3.003)		
**RA population, n (%)**					1615 (100)			1615 (100)			1615 (100)			1669 (100)
	Effect analysis by one linear model, HR (95% CI)			1.000 (0.934-1.070)			0.984 (0.875-1.106)			0.980 (0.859-1.119)			1.073 (0.876-1.314)	
	**Effect analysis by 2-fold piecewise model, HR (95% CI)**													
		Sleep duration＜inflection point	0.845^f^ (0.739-0.965)			0.753^d^ (0.608-0.933)			0.794 (0.619-1.019)			0.985 (0.782-1.241)		
		Sleep duration≥inflection point	1.133^f^ (1.017-1.262)			1.215^f^ (1.014-1.457)			1.161 (0.940-1.434)			1.893 (0.897-3.995)		
		Effect difference	1.341^d^ (1.091-1.649)			1.614^d^ (1.151-2.264)			1.462 (0.984-2.173)			1.921 (0.807-4.575)		
**HUA population, n (%)**					5538 (100)			5538 (100)			5538 (100)			5725 (100)
	Effect analysis by one linear model, HR (95% CI)			0.979 (0.937-1.023)			0.978 (0.913-1.049)			1.042 (0.946-1.149)			0.988 (0.881-1.108)	
	**Effect analysis by 2-fold piecewise model, HR (95% CI)**													
		Sleep duration＜inflection point	0.852^f^ (0.797-0.911)			0.852^d^ (0.766-0.947)			0.942 (0.843-1.053)			0.743^f^ (0.633-0.871)		
		Sleep duration≥inflection point	1.216^f^ (1.113-1.328)			1.212^d^ (1.055-1.393)			1.731^f^ (1.300-2.305)			1.546^f^ (1.264-1.891)		
		Effect difference	1.427^f^ (1.250-1.629)			1.423^d^ (1.154-1.755)			1.838^f^ (1.300-2.597)			2.083^f^ (1.527-2.840)		

^a^The model used for the threshold effect analysis was adjusted for age, sex, race, educational level, marital status, total energy intake, BMI, physical activity, alcohol consumption, smoking, hypertension, and diabetes.

^b^CVD: cardiovascular disease.

^c^HR: hazard ratio.

^d^*P*<.01.

^e^*P*<.001.

^f^*P*<.05.

Below the inflection point, a significantly decreased HR for all-cause mortality was detected, whereas a significantly increased HR for all-cause, CVD-cause, and diabetes-cause mortality was detected above the inflection point. In the RA population, the detected inflection point of sleep duration for all-cause and CVD-cause mortality was 6.5 hours. Below the inflection point, a significantly decreased HR for all-cause and CVD-cause mortality was detected. Above the inflection point, a significantly increased HR for all-cause and CVD-cause mortality was detected. In the HUA population, the inflection point of sleep duration for all-cause, CVD-cause, cancer-cause, and diabetes-cause mortality in model 2 was 7.5 hours, 7.5 hours, 9 hours, and 7.5 hours, respectively. Below the inflection point, a significantly decreased HR for all-cause, CVD-cause, and diabetes-cause mortality was detected. Above the inflection point, a significantly increased HR for all-cause, CVD-cause, cancer-cause, and diabetes-cause mortality was detected.

## Discussion

### Principal Findings

In this study, we found that increased sleep duration was associated with decreased BMI, obesity, WC, abdominal obesity, OA, and RA but increased BMD. The nonlinear analysis indicated that sleep duration caused a saturation effect on obesity, abdominal obesity, and HUA. In addition, a significant threshold effect of sleep duration on BMD; OA; RA; and all-cause, CVD-cause, cancer-cause, and diabetes-cause mortality was found.

Sleep is an indispensable physiological activity for human beings that is closely associated with physical and mental health and the incidence and development of various diseases. Despite the critical significance of healthy sleep, sleep disorders have gradually increased over the past 2 decades, with more than a third of adults and 57% of teenagers in the United States experiencing insomnia [20]. With aggravating the worrying sleep dilemma, accumulating interests have been aroused in exploring sleep’s physiological and pathological role in human beings. According to the International Classification of Sleep Disorders published by the American Academy of Sleep Medicine, the diagnosis of sleep disorders can be divided into 7 types, including insomnia, sleep-related breathing disorders, central disorders of hypersomnolence, circadian rhythm sleep-wake disorders, parasomnias, sleep-related movement disorders, and other sleep disorders [21]. These sleep disorders would significantly impair sleep duration, which is essential to maintain mental health [22,23].

Many studies have investigated the association between obesity and sleep duration, with controversial conclusions achieved. A negative association between obesity and sleep duration has been detected in some studies [24-27], whereas none of these studies have a sample size of>10,000 participants. In addition, Kripke et al [28] reported a U-shaped association between BMI and sleep duration in 636,095 female participants in the United States, with an inflection point of sleep duration (7 to 8 hours). In comparison, no significant association between BMI and sleep duration was detected in 480,841 male participants. To the best of our knowledge, we are the first to find the saturation effect of sleep duration on obesity-related outcomes, indicating a negative association between obesity and sleep duration when sleep duration is <8 hours. Some epidemiological studies have pointed out that sleep duration may cause obesity and endocrine changes such as decreased glucose tolerance and insulin sensitivity, increased evening concentrations of cortisol, increased levels of ghrelin, decreased levels of leptin, and increased hunger and appetite [29,30]. Although only a few experimental studies have explored the underlying mechanism behind this phenomenon, it could be hypothesized that the underlying mechanism is complex and works in multiple networks.

In this study, we have found a n-shaped association between BMD and sleep duration, with the inflection point of 7.5 hours sleep duration detected. On the basis of the NHANES program 2017-2018, Lee et al [6] found that the osteoporosis incidence of female participants aged >50 years with a sleep duration of <5 hours was significantly higher than that of normal participants (OR 7.35) in the NHANES program 2005-2010, which was almost consistent with the findings of Shiao et al [31]. In addition, Tang et al [32] found that participants with shorter or longer sleep durations showed lower BMD than those with normal sleep duration (7 to 9 hours), which was consistent with our findings. It should not be ignored that some studies reported no significant association between sleep duration and BMD [33] and osteoporosis [34]. These epidemiological studies revealed that sleep duration seems to be a double-edged sword in affecting the change of BMD. However, more experimental evidence should be provided to demonstrate this phenomenon. Swanson et al [35] found that the level of P1NP, a biomarker of bone formation, decreased when the sleep circadian rhythm was disrupted, which was more pronounced among younger people and indicated a detrimental effect of sleep disorders on normal bone turnover. In addition, it was reported that growth hormone might be an important effective agent involving sleep duration affecting bone mass [36]. In general, many epidemiological studies have demonstrated the detrimental effect of sleep disorders on BMD, although exploring specific molecular mechanisms needs further work.

UA is the terminal metabolite of purine compounds, of which approximately 80% are endogenous and 20% are derived from exogenous food rich in purine or nucleic acid proteins [37]. Under normal physiological conditions, the production and catabolism of UA are in dynamic homeostasis, mainly regulated by liver synthesis and excretion from or absorption in the kidney and intestine [38]. Excessive serum UA, HUA, was reported to be closely associated with various diseases, including gout, chronic kidney disease, and CVD [39,40]. A few epidemiological studies have investigated the association between sleep duration and UA or HUA. Lee et al [41] found a U-shaped association between sleep duration and UA in Korean women, indicating that female individuals with a sleep duration of 7 to 8 hours showed the lowest UA level. In addition, many related studies have reported the negative association that increased sleep duration was significantly associated with a lower risk of HUA [42-44]. However, in this study, we found a threshold effect between sleep duration and HUA, indicating a positive association between HUA and sleep duration when sleep duration was <5 hours. This finding was different from that of previous studies. Owing to the lack of experimental evidence and controversy regarding epidemiological studies, it could be challenging to draw an affirmative conclusion related to the association between sleep duration and UA level up till now.

OA is one of the most common musculoskeletal diseases, seriously impairing the health and social functioning of the US population. Recent epidemiological studies have investigated the association between sleep duration and OA. On the basis of the Korea NHANES program, Park et al [45] found a U-shaped association between sleep duration and OA in the older woman population (aged >50 years), with a lower incidence of OA occurring in the group reporting a 7 to 8 hours sleep duration. Ni et al [46] found that the increased OA incidence was positively correlated with a shorter sleep duration. These studies were conducted within a specific population in Korea. At the same time, there were few studies to investigate the association between sleep duration and OA in the general US population. What we performed in this study tried to answer this question, and we found a threshold effect of sleep duration on OA incidence in the US population. The OA incidence increased with the increase in sleep duration; however, this synergistically positive association disappeared when sleep duration increased to 8 hours. It is worth noting that Ni et al [46] found that patients with OA aged >60 years in the NHANES program (2011 to 2018) experienced a higher incidence of sleep trouble [46]. These studies and our findings have demonstrated a positive association between sleep duration and OA incidence, although the causal association between them is yet to be clarified. It is well known that progressing OA pathology into the advanced stage causes severe pain and impairs the patient’s function and sleep quality [47,48]. Therefore, the more profound association between sleep duration and OA, such as a causal relationship, should be verified by more prospective clinical studies.

As an autoimmune disease, RA is caused by multiple factors, including genetic susceptibility and environmental factors [49]. Kim et al [50] found that the RA incidence in the Korean population decreased with an increase in sleep duration when sleep duration was <6 hours. They also found that severe RA pain was closely associated with sleep duration when it was <6 hours [50]. Meanwhile, Wu et al [51] found that shorter sleep duration, <6 hours, and poor sleep quality were positively associated with RA incidence. This study found that increased sleep duration was negatively associated with RA. A threshold effect (inflection point: 6.5 hours) was detected in the association analysis between sleep duration and RA. The association we detected between sleep duration (<6.5 hours) and RA was almost consistent with the aforementioned studies. It is worth noting that we were the first to find that excessive sleep duration (>6.5 hours) was a risk factor for RA. However, the causal relationship between sleep duration and RA was challenging to be verified because the pain symptom was one of the main characteristics of RA, which could also cause detrimental influences on sleep quality. Notably, Gao et al [52] performed a Mendelian randomization study to investigate the causal relationship between sleep traits and RA. They found no association between sleep duration and frequent insomnia, any insomnia, sleep duration, or snoring, although the casual effect of shorter sleep duration (<6 hours) on RA was detected through inverse variance weighted and weighted median methods, which provided further evidence to support our findings. Despite this accumulating epidemiological evidence and continuous insight into the pathogenesis of RA, research on how sleep duration or traits affect the occurrence and development of RA still needs to be completed. Further experimental studies focusing on this perspective may yield a new understanding of the pathogenesis of RA.

Many epidemiological studies have widely investigated the association between sleep duration and mortality [2,53,54]. The U-shaped association (inflection point: 6.5 hours) detected in this study between sleep duration and all-cause, CVD-cause, and cancer-cause mortality was first detected in the general US population, which was consistent with a previous study involving a specific population, such as older or woman populations [55-57]. With the change in dietary structure, diabetes has gradually become one of the most common aging diseases, causing serious detrimental effects on health and longevity [58,59]. However, mortality, mainly or partly because of diabetes, has been less investigated, especially for investigating the effect of life behaviors on diabetes-cause mortality in the general or specific population. Hou et al [60] investigated the association between diabetes-cause mortality and carbohydrate intake behaviors and found that low-quality carbohydrates at dinner were positively associated with diabetes-cause mortality. We first explored the association between sleep duration and diabetes-cause mortality, with a U-shaped association detected in the general US population. We also detected a similar U-shaped association between sleep duration and diabetes-cause mortality in the OA and HUA populations. These findings provide a strong epidemiological evidence that lifestyle behaviors, such as dietary and sleep behaviors, may have played an essential role in affecting poor diabetes-related outcomes. A large amount of epidemiological evidence have been provided to demonstrate that shorter or excessive sleep duration causes a detrimental effect on mortality outcomes. Unlike previous studies, we are the first to identify a threshold effect of sleep duration on mortality in the OA, RA, and HUA populations. This discovery offers new clinical evidence about the impact of sleep duration on mortality outcomes within these specific populations, highlighting the potential benefits of maintaining an appropriate sleep duration for individuals in these groups. There are some potential explanations from the perspective of mechanisms to clarify the negative association between shorter sleep duration and mortality, including increased cortisol secretion, altered growth hormone metabolism, inflammation activation, and changes in circulating leptin and ghrelin [2,61,62]. Although no such hypothesis has been provided to clarify the detrimental effect of excessive sleep duration on mortality in the general or specific population, further experimental studies must be conducted.

### Study Limitations

Some limitations should be addressed when generalizing the conclusions. First, as a cross-sectional study, this study highlighted the association between sleep duration and various outcomes; however, it was not possible to infer the causal association between sleep duration and these outcomes. Mendelian randomization analysis could be an alternative method to investigate their causal association, which may be conducted to further determine their causal association in the US population. Second, the diagnosis of OA and RA was defined through a questionnaire survey method, which may lead to misdiagnosing or missed diagnosis of some participants and impair the evidence level of our conclusion. Third, although we included various covariates for adjustment in the analysis, there must be missing covariates associated with the outcomes analyzed in this study. For example, genetic factors, family history, and environmental exposures have been considered as the risk factors for some diseases, such as RA and obesity. Owing to the limitation of data availability, it was difficult to include these individual information as covariates. Furthermore, the sleep duration information of children was absent in the NHANES program. Therefore, we should be careful when generalizing the conclusions to specific populations. In addition, the quality of sleep is an important factor influencing the health status; however, the lack of this individual information in the NHANES program makes it difficult to analyze the role of sleep quality on individual health, which should be investigated in future studies. In general, more prospective and related experimental studies or Mendelian randomization studies are needed to further validate the conclusions of this study.

### Conclusions

In our study, which included a total of 54,664 participants across 8 survey cycles of the NHANES program, we observed that longer sleep duration was associated with decreased BMI, obesity, WC, abdominal obesity, OA, and RA but increased BMD. Furthermore, we found that sleep duration caused a saturation effect on obesity, abdominal obesity, and HUA. In addition, a significant threshold effect of sleep duration on BMD; OA; RA; and all-cause, CVD-cause, cancer-cause, and diabetes-cause mortality was found. In conclusion, this study provided further evidence about the double-edged sword effect of sleep duration on health, and proper sleep duration plays a critical role in maintaining good health status. These findings suggested that ensuring adequate sleep duration should be an integral part of public health programs.

## Abbreviations

BMD: bone mineral density
CVD: cardiovascular disease
HR: hazard ratio
HUA: hyperuricemia
NHANES: National Health and Nutrition Examination Survey
OA: osteoarthritis
OR: odds ratio
RA: rheumatoid arthritis
UA: uric acid
WC: waist circumference

## Data Availability

All data used in this study are available on the National Health and Nutrition Examination Survey (NHANES) website [63].

## References

[1] Knutson KL, Van Cauter E (2008). Associations between sleep loss and increased risk of obesity and diabetes. Ann N Y Acad Sci.

[2] Cappuccio FP, D'Elia L, Strazzullo P, Miller MA (2010). Sleep duration and all-cause mortality: a systematic review and meta-analysis of prospective studies. Sleep.

[3] Svensson T, Saito E, Svensson AK, Melander O, Orho-Melander M, Mimura M, Rahman S, Sawada N, Koh WP, Shu XO, Tsuji I, Kanemura S, Park SK, Nagata C, Tsugane S, Cai H, Yuan JM, Matsuyama S, Sugawara Y, Wada K, Yoo KY, Chia KS, Boffetta P, Ahsan H, Zheng W, Kang D, Potter JD, Inoue M (2021). Association of sleep duration with all- and major-cause mortality among adults in Japan, China, Singapore, and Korea. JAMA Netw Open.

[4] Wang C, Bangdiwala SI, Rangarajan S, Lear SA, AlHabib KF, Mohan V, Teo K, Poirier P, Tse LA, Liu Z, Rosengren A, Kumar R, Lopez-Jaramillo P, Yusoff K, Monsef N, Krishnapillai V, Ismail N, Seron P, Dans AL, Kruger L, Yeates K, Leach L, Yusuf R, Orlandini A, Wolyniec M, Bahonar A, Mohan I, Khatib R, Temizhan A, Li W, Yusuf S (2019). Association of estimated sleep duration and naps with mortality and cardiovascular events: a study of 116 632 people from 21 countries. Eur Heart J.

[5] Hirsch JK, Altier HR, Offenbächer M, Toussaint L, Kohls N, Sirois FM (2021). Positive psychological factors and impairment in rheumatic and musculoskeletal disease: do psychopathology and sleep quality explain the linkage?. Arthritis Care Res (Hoboken).

[6] Lee C-L, Tzeng H-E, Liu W-J, Tsai C-H (2021). A cross-sectional analysis of the association between sleep duration and osteoporosis risk in adults using 2005-2010 NHANES. Sci Rep.

[7] Martínez-Rodríguez A, Rubio-Arias JÁ, Ramos-Campo DJ, Reche-García C, Leyva-Vela B, Nadal-Nicolás Y (2020). Psychological and sleep effects of tryptophan and magnesium-enriched Mediterranean diet in women with fibromyalgia. Int J Environ Res Public Health.

[8] Zou L, Yeung A, Quan X, Boyden SD, Wang H (2018). A systematic review and meta-analysis of mindfulness-based (Baduanjin) exercise for alleviating musculoskeletal pain and improving sleep quality in people with chronic diseases. Int J Environ Res Public Health.

[9] Dashti HS, Ordovás JM (2021). Genetics of sleep and insights into its relationship with obesity. Annu Rev Nutr.

[10] St-Onge M-P (2017). Sleep-obesity relation: underlying mechanisms and consequences for treatment. Obes Rev.

[11] Reutrakul S, Van Cauter E (2018). Sleep influences on obesity, insulin resistance, and risk of type 2 diabetes. Metabolism.

[12] Petrov ME, Long DL, Grandner MA, MacDonald LA, Cribbet MR, Robbins R, Cundiff JM, Molano JR, Hoffmann CM, Wang X, Howard G, Howard VJ (2020). Racial differences in sleep duration intersect with sex, socioeconomic status, and U.S. geographic region: the REGARDS study. Sleep Health.

[13] Troxel WM, Lee L, Hall M, Matthews KA (2014). Single-parent family structure and sleep problems in Black and White adolescents. Sleep Med.

[14] Di H, Guo Y, Daghlas I, Wang L, Liu G, Pan A, Liu L, Shan Z (2022). Evaluation of sleep habits and disturbances among US adults, 2017-2020. JAMA Netw Open.

[15] NCHS Ethics Review Board (ERB) approval. Centers for Disease Control and Prevention.

[16] Njeh CF, Fuerst T, Hans D, Blake GM, Genant HK (1999). Radiation exposure in bone mineral density assessment. Appl Radiat Isot.

[17] March LM, Schwarz JM, Carfrae BH, Bagge E (1998). Clinical validation of self-reported osteoarthritis. Osteoarthritis Cartilage.

[18] Gregg EW, Cheng YJ, Srinivasan M, Lin J, Geiss LS, Albright AL, Imperatore G (2018). Trends in cause-specific mortality among adults with and without diagnosed diabetes in the USA: an epidemiological analysis of linked national survey and vital statistics data. Lancet.

[19] Tippmann S (2015). Programming tools: adventures with R. Nature.

[20] Mattingly SM (2021). Sleep, health, productivity, and the double-edged sword of technology. XRDS Crossroads ACM Mag Stud.

[21] Sateia MJ (2014). International classification of sleep disorders-third edition: highlights and modifications. Chest.

[22] Matricciani LA, Olds TS, Blunden S, Rigney G, Williams MT (2012). Never enough sleep: a brief history of sleep recommendations for children. Pediatrics.

[23] Matricciani L, Bin YS, Lallukka T, Kronholm E, Dumuid D, Paquet C, Olds T (2017). Past, present, and future: trends in sleep duration and implications for public health. Sleep Health.

[24] Gangwisch JE, Malaspina D, Boden-Albala B, Heymsfield SB (2005). Inadequate sleep as a risk factor for obesity: analyses of the NHANES I. Sleep.

[25] Eisenmann JC, Ekkekakis P, Holmes M (2006). Sleep duration and overweight among Australian children and adolescents. Acta Paediatr.

[26] Ko GT, Chan JC, Chan AW, Wong PT, Hui SS, Tong SD, Ng S, Chow F, Chan CL (2007). Association between sleeping hours, working hours and obesity in Hong Kong Chinese: the 'better health for better Hong Kong' health promotion campaign. Int J Obes (Lond).

[27] Cappuccio FP, Taggart FM, Kandala N-B, Currie A, Peile E, Stranges S, Miller MA (2008). Meta-analysis of short sleep duration and obesity in children and adults. Sleep.

[28] Kripke DF, Garfinkel L, Wingard DL, Klauber MR, Marler MR (2002). Mortality associated with sleep duration and insomnia. Arch Gen Psychiatry.

[29] Zimberg IZ, Dâmaso A, Del Re M, Carneiro AM, de Sá Souza H, de Lira FS, Tufik S, de Mello MT (2012). Short sleep duration and obesity: mechanisms and future perspectives. Cell Biochem Funct.

[30] Felső R, Lohner S, Hollódy K, Erhardt É, Molnár D (2017). Relationship between sleep duration and childhood obesity: systematic review including the potential underlying mechanisms. Nutr Metab Cardiovasc Dis.

[31] Shiao Y-C, Chen W-T, Chen W-L (2021). Association of short sleep duration and trabecular bone score. Sci Rep.

[32] Tang Y, Wang S, Yi Q, Xia Y, Geng B (2021). Sleep pattern and bone mineral density: a cross-sectional study of National Health and Nutrition Examination Survey (NHANES) 2017-2018. Arch Osteoporos.

[33] Marques EA, Figueiredo P, Gudnason V, Lang T, Sigurdsson G, Sigurdsson S, Aspelund T, Siggeirsdottir K, Launer L, Eiriksdottir G, Harris TB (2017). Associations of 24-hour sleep duration and CT-derived measurements of muscle and bone: the AGES-Reykjavik study. Exp Gerontol.

[34] Moradi S, Shab-Bidar S, Alizadeh S, Djafarian K (2017). Association between sleep duration and osteoporosis risk in middle-aged and elderly women: a systematic review and meta-analysis of observational studies. Metabolism.

[35] Swanson CM, Shea SA, Wolfe P, Cain SW, Munch M, Vujovic N, Czeisler CA, Buxton OM, Orwoll ES (2017). Bone turnover markers after sleep restriction and circadian disruption: a mechanism for sleep-related bone loss in humans. J Clin Endocrinol Metab.

[36] Stich FM, Huwiler S, D'Hulst G, Lustenberger C (2022). The potential role of sleep in promoting a healthy body composition: underlying mechanisms determining muscle, fat, and bone mass and their association with sleep. Neuroendocrinology.

[37] Maiuolo J, Oppedisano F, Gratteri S, Muscoli C, Mollace V (2016). Regulation of uric acid metabolism and excretion. Int J Cardiol.

[38] Lima WG, Martins-Santos ME, Chaves VE (2015). Uric acid as a modulator of glucose and lipid metabolism. Biochimie.

[39] Prasad Sah OS, Qing YX (2015). Associations between hyperuricemia and chronic kidney disease: a review. Nephrourol Mon.

[40] Zhang S, Wang Y, Cheng J, Huangfu N, Zhao R, Xu Z, Zhang F, Zheng W, Zhang D (2019). Hyperuricemia and cardiovascular disease. Curr Pharm Des.

[41] Lee Y-C, Son D-H, Kwon Y-J (2020). U-shaped association between sleep duration, C-reactive protein, and uric acid in Korean women. Int J Environ Res Public Health.

[42] Yu X, Gong S, Chen J, Zhang H, Shen Z, Gu Y, Lv S, Zhang D, Wang Y, Ding X, Zhang X (2021). Short sleep duration increases the risk of hyperuricemia among Chinese adults: findings from the China Health and Nutrition Survey. Sleep Med.

[43] An Y, Li X, Ouyang F, Xiao S (2022). Association between nocturnal sleep duration and the risk of hyperuricemia among Chinese government employees: a cross-sectional study. Front Public Health.

[44] Papandreou C, Babio N, Díaz-López A, Martínez-González MA, Becerra-Tomas N, Corella D, Schröder H, Romaguera D, Vioque J, Alonso-Gómez ÁM, Wärnberg J, Martínez AJ, Serra-Majem L, Estruch R, Muñoz-Garach A, Lapetra J, Pintó X, Tur J, Garcia-Rios A, Bueno-Cavanillas A, Delgado-Rodríguez M, Matía-Martín P, Daimiel L, M Sánchez V, Vidal J, Vázquez C, Ros E, Ruiz-Canela M, Bulló M, Sorli J, Quifer M, Colom A, Oncina-Canovas A, T Sierra L, Barón-López J, Pérez-Farinós N, Abete I, Sanchez-Villegas A, Casas R, F García JC, Santos-Lozano J, Corbella E, Del M Bibiloni M, Diez-Espino J, Asensio E, Torras L, Morey M, Compañ-Gabucio L, S Lete I, Cenoz-Osinaga J, Castañer O, Salas-Salvadó J (2019). Sleep duration is inversely associated with serum uric acid concentrations and uric acid to creatinine ratio in an elderly mediterranean population at high cardiovascular risk. Nutrients.

[45] Park H-M, Kwon Y-J, Kim H-S, Lee Y-J (2019). Relationship between sleep duration and osteoarthritis in middle-aged and older women: a nationwide population-based study. J Clin Med.

[46] Ni J, Zhou W, Cen H, Chen G, Huang J, Yin K, Sui C (2022). Evidence for causal effects of sleep disturbances on risk for osteoarthritis: a univariable and multivariable Mendelian randomization study. Osteoarthritis Cartilage.

[47] Turk DC, Cohen MJ (2010). Sleep as a marker in the effective management of chronic osteoarthritis pain with opioid analgesics. Semin Arthritis Rheum.

[48] Sasaki E, Tsuda E, Yamamoto Y, Maeda S, Inoue R, Chiba D, Okubo N, Takahashi I, Nakaji S, Ishibashi Y (2014). Nocturnal knee pain increases with the severity of knee osteoarthritis, disturbing patient sleep quality. Arthritis Care Res (Hoboken).

[49] Costenbader KH, Chang S-C, De Vivo I, Plenge R, Karlson EW (2008). Genetic polymorphisms in PTPN22, PADI-4, and CTLA-4 and risk for rheumatoid arthritis in two longitudinal cohort studies: evidence of gene-environment interactions with heavy cigarette smoking. Arthritis Res Ther.

[50] Kim J-H, Park E-C, Lee KS, Lee Y, Shim S, Kim J, Chon D, Lee S-G (2016). Association of sleep duration with rheumatoid arthritis in Korean adults: analysis of seven years of aggregated data from the Korea National Health and Nutrition Examination Survey (KNHANES). BMJ Open.

[51] Wu W, Yang J, Gu Y, Chen X, Tan X (2020). Dose-response relationship between sleep and rheumatoid arthritis. Am J Health Behav.

[52] Gao R-C, Sang N, Jia C-Z, Zhang M-Y, Li B-H, Wei M, Wu G-C (2022). Association between sleep traits and rheumatoid arthritis: a mendelian randomization study. Front Public Health.

[53] Irwin MR, Olmstead R, Carroll JE (2016). Sleep disturbance, sleep duration, and inflammation: a systematic review and meta-analysis of cohort studies and experimental sleep deprivation. Biol Psychiatry.

[54] Yin J, Jin X, Shan Z, Li S, Huang H, Li P, Peng X, Peng Z, Yu K, Bao W, Yang W, Chen X, Liu L (2017). Relationship of sleep duration with all-cause mortality and cardiovascular events: a systematic review and dose-response meta-analysis of prospective cohort studies. J Am Heart Assoc.

[55] Xiao Q, Blot WJ, Matthews CE (2019). Weekday and weekend sleep duration and mortality among middle-to-older aged White and Black adults in a low-income southern US cohort. Sleep Health.

[56] Gupta K, Nagalli S, Kalra R, Gupta R, Mahmood S, Jain V, Zhou W, Prabhu SD, Bajaj NS (2021). Sleep duration, baseline cardiovascular risk, inflammation and incident cardiovascular mortality in ambulatory U.S. Adults: national health and nutrition examination survey. Am J Prev Cardiol.

[57] Kabat GC, Xue X, Kamensky V, Zaslavsky O, Stone KL, Johnson KC, Wassertheil-Smoller S, Shadyab AH, Luo J, Hale L, Qi L, Cauley JA, Brunner RL, Manson JE, Rohan TE (2018). The association of sleep duration and quality with all-cause and cause-specific mortality in the Women's Health Initiative. Sleep Med.

[58] Furman D, Campisi J, Verdin E, Carrera-Bastos P, Targ S, Franceschi C, Ferrucci L, Gilroy DW, Fasano A, Miller GW, Miller AH, Mantovani A, Weyand CM, Barzilai N, Goronzy JJ, Rando TA, Effros RB, Lucia A, Kleinstreuer N, Slavich GM (2019). Chronic inflammation in the etiology of disease across the life span. Nat Med.

[59] Lin D, Xiao M, Zhao J, Li Z, Xing B, Li X, Kong M, Li L, Zhang Q, Liu Y, Chen H, Qin W, Wu H, Chen S (2016). An overview of plant phenolic compounds and their importance in human nutrition and management of type 2 diabetes. Molecules.

[60] Hou W, Han T, Sun X, Chen Y, Xu J, Wang Y, Yang X, Jiang W, Sun C (2022). Relationship between carbohydrate intake (quantity, quality, and time eaten) and mortality (total, cardiovascular, and diabetes): assessment of 2003-2014 national health and nutrition examination survey participants. Diabetes Care.

[61] Spiegel K, Tasali E, Penev P, Van Cauter E (2004). Brief communication: sleep curtailment in healthy young men is associated with decreased leptin levels, elevated ghrelin levels, and increased hunger and appetite. Ann Intern Med.

[62] Taheri S, Lin L, Austin D, Young T, Mignot E (2004). Short sleep duration is associated with reduced leptin, elevated ghrelin, and increased body mass index. PLoS Med.

[63] National Health and Nutrition Examination Survey. Centers for Disease Control and Prevention.

